# Serum Erythropoietin and Ischemic-Modified Albumin Levels in Adolescents with Obsessive–Compulsive Disorder

**DOI:** 10.1007/s12031-024-02247-x

**Published:** 2024-07-12

**Authors:** Masum Öztürk, Fatma Subaşı Turgut, Davut Akbalık, Mustafa Erhan Demirkıran, İbrahim Kaplan

**Affiliations:** 1 Department of Child and Adolescent Psychiatry, Faculty of Medicine, Dicle University, Diyarbakır, Turkey; 2 Department of Biochemistry, Faculty of Medicine, Dicle University, Diyarbakır, Turkey

**Keywords:** Erythropoietin, Ischemic-modified albumin, Oxidative stress, Obsessive–compulsive disorder

## Abstract

Erythropoietin (EPO) has neuroprotective effects by increasing oxidative stress resistance and stabilizing redox balance. Ischemic-modified albumin (IMA) is a product of protein oxidation, and recent evidence suggests that IMA can be used as an indicator of oxidative damage. This study aimed to investigate serum EPO and IMA levels in obsessive–compulsive disorder (OCD) patients and to investigate the relationship between EPO and IMA levels and clinical variables such as disease duration and disease severity. A total of 68 adolescents (11–18 years old), including 35 OCD patients (18 males/17 females) and 33 healthy controls (14 males/19 females) without comorbid disorders matched for age, gender, and BMI, were included in the study. The enzyme-amplified chemiluminescence technique determined serum EPO levels, and serum IMA levels were determined by the spectrophotometric method. Serum EPO levels were lower in OCD patients compared to healthy controls (*p* = 0.002; *Z* =  − 3.123), and serum IMA levels (ABSU) were significantly higher in the OCD group (*p* = 0.005). A significant positive correlation was found between IMA levels and the duration of OCD symptoms (*p* = 0.015, *r* = 0.409). The study’s findings contribute to the growing body of evidence implicating inflammatory and oxidative processes in the pathogenesis of OCD. The potential of EPO and IMA levels as diagnostic biomarkers for OCD aligns with the ongoing efforts to identify reliable biological markers for the disorder. The positive correlation of IMA levels with the duration of OCD shows the importance of early detection of oxidative damage.

## Introduction

Obsessive–compulsive disorder (OCD) is a chronic psychiatric disorder characterized by intrusive thoughts and repetitive behaviors known as obsessions and compulsions (Goodman et al. 2021). OCD is one of the most common mental disorders and is a significant contributor to the global burden of disease. Research indicates that the lifetime prevalence of OCD is 2–3% and that this disorder is associated with significant comorbidity and morbidity (Stein et al. 2019). The age at onset of OCD varies widely, with symptoms appearing in very early childhood through to adulthood. In 30 to 50% of individuals with OCD, symptoms begin in childhood, often before the age of 10 (Pauls et al. 2014). The etiology of OCD involves the interaction of many genetic, inflammatory, and environmental factors. Understanding the pathophysiology of OCD is crucial for developing effective treatment strategies (Alsheikh and Alsheikh 2021). Neuroinflammation has recently emerged as a potential factor contributing to OCD. Accumulating evidence suggests that oxidative stress (OS) may contribute to the etiology of OCD through neuronal damage (Alici et al. 2016; Gerentes et al. 2019).

OS leads to lipid peroxidation, causing damage to polyunsaturated fatty acids, which are particularly vulnerable to ROS. This process generates aldehydes that can interact with nucleic acids and proteins, ultimately leading to cellular structural and functional impairment, potentially surpassing the cell’s repair capacity and triggering apoptosis (Ayala et al. 2014; Tampa et al. 2022). Studies have also demonstrated that high levels of OS critically increase neuronal cell death, while inhibition of OS by overexpression of antioxidant proteins reduces neuronal cell death (Cho et al. 2019). Clinical studies have shown that the oxidative stress index is elevated, and antioxidant defense mechanisms are decreased in OCD (Kandemir et al. 2013). Understanding these mechanisms could provide valuable insights for developing targeted therapeutic interventions.

Erythropoietin (EPO) is considered the primary growth and differentiation factor in erythroid cells, and the best-known function of this glycoprotein is the regulation of erythrocyte production (Vittori et al. 2021). EPO is produced by the kidney and acts primarily on red blood cells. However, EPO also has hormonal effects in other organs, including the brain, heart, and retina, in addition to hematopoietic tissues (Lai et al. 2022). EPO has important effects for repairing brain damage and neurodevelopment. Its anti-inflammatory, anti-excitotoxic, antioxidant, and anti-apoptotic effects and regenerative properties in the brain are responsible for its neuroprotective effects (Juul et al. 2018). As observed in in vitro and ex vivo studies, EPO exhibits neuroprotective effects by increasing OS resistance and stabilizing the redox equilibrium (Ottolenghi et al. 2021). Studies have also shown that EPO has been effective in reducing anxiety-related behavior and exploratory impairments in models of mood disorders and hypoxia, further underlining its potential role in psychiatric symptoms (Larpthaveesarp et al. 2021). EPO levels have been investigated in generalized anxiety disorder in adults and attention deficit hyperactivity disorder in children (Gungor et al. 2021; Kurutas 2023). In both disorders, serum EPO levels were reported to be lower than those of healthy controls. There are also recent studies investigating that EPO levels may be associated with suicidality in patients with depression (Lee et al. 2021).

Decreased albumin production or changes in its functional activity can affect oncotic blood pressure and interfere with hormone, fatty acid, and metal transport. In conditions such as OS and acidosis, changes occur in the biochemical structure of albumin. Ischemic-modified albumin (IMA) is produced with reduced capacity for certain metals (nickel, cobalt, copper) (Shevtsova et al. 2021). The advantages of using IMA as a biomarker are that it can be detected earlier in ischemic and OS conditions, and its serum levels are simple and practical (Shevtsova et al. 2021). Studies indicate that changes in IMA levels may indicate neuronal damage in central nervous system (CNS) pathologies and damage to the blood–brain barrier in psychiatric disorders (Shevtsova et al. 2021). IMA, which is reported to be associated with OS and inflammation, was first studied in cardiovascular diseases and has been associated with many pathological conditions or diseases other than cardiovascular diseases. IMA is, therefore, thought to be a marker for diseases that are both ischemia-mediated and OS-related (Seshadri Reddy et al. 2018). IMA levels have also been investigated in CNS diseases in recent years, and it has been reported that IMA levels are elevated in the peripheral circulation during the prodromal period of Alzheimer’s disease (Du et al. 2019). IMA levels have been previously investigated in autism spectrum disorder in children and bipolar disorder in adults, and IMA levels have been reported to increase in patient groups (Ceylan et al. 2021; Korkmaz et al 2023). It has been reported that oxidative stress may be activated in these disorders, and early detection of IMA as an indicator of oxidative stress may be clinically valuable.

As far as we know, no academic study has examined the serum levels of EPO and IMA in patients with OCD. Therefore, our study aimed to compare serum EPO and IMA levels in pediatric patients diagnosed with OCD and a demographically matched, healthy control group. In addition, we aimed to assess whether there is a significant relationship between EPO and IMA levels and the severity of OCD symptoms in affected individuals. This research aims to contribute to the advancement of our understanding of the pathophysiological mechanisms associated with OCD and increase our knowledge of diagnosis and therapeutic intervention.

## Methods

### Participants

This cross-sectional study was conducted in the Child and Adolescent Psychiatry Outpatient Clinic of Dicle University Faculty of Medicine. The sample size was calculated as 62 participants (31 participants for each group) with a 5% significance level and 1-*β* = 0.85 (85%) power. To increase the study’s power, 68 participants were included. A similar number of participants was included in another study previously conducted with the OCD group (Ozkan et al. 2021). The study involved 35 patients between 11 and 18 years old diagnosed with OCD, along with 33 healthy controls in the same age group without any psychiatric or medical illnesses. First of all, the patients who applied to the clinic where the study was conducted and diagnosed with OCD were referred to the researchers. Participants whose OCD diagnosis was confirmed by a clinical interview conducted by the researcher and who met the inclusion and exclusion criteria of the study were then administered the CY-BOCS by clinicians, and participants with a CY-BOCS total score > 7 were included in the OCD group. Participants with comorbid psychiatric disorders (anxiety disorder, mood disorders, autism, tic disorder, schizophrenia, mental retardation, attention deficit hyperactivity disorder (ADHD), eating disorder) accompanying the OCD group were excluded. K-SADS-DSM-5-PL (Ünal et al. 2019) was applied to confirm the diagnosis of OCD and screen for comorbid psychiatric disorders. In the control group, participants with similar sociodemographic characteristics (age, gender, BMI) to the participants in the OCD group, aged between 11 and 18 years, who presented with academic failure, attention problems, adolescence period problems, and whose clinical evaluation, scales applied, and biochemical examinations did not reveal any problems were referred to the researcher. As a result of the K-SADS-DSM-5-PL (Ünal et al. 2019), participants who were not currently or in the past diagnosed with any psychiatric disorder, had no previous psychiatric admission or psychotropic drug use, and whose BMI was within the normal range constituted the healthy control group. In addition, participants with known chronic medical diseases (e.g., epilepsy, endocrinologic disease, kidney disease), obesity, inflammatory or autoimmune diseases, history of alcohol and substance abuse, taking additional food supplements such as vitamins and fish oil, active infection, or history of infection in the last 1 month, use of drugs that may affect the immune system in the last 6 months, and use of anti-inflammatory drugs in the last month were not included in both OCD and control groups. After all participants and parents were informed about the study, written and verbal informed assent was obtained from parents and children. Before starting the study, ethical approval was obtained from the local ethics committee (Dicle University Faculty of Medicine Non-Interventional Clinical Research Ethics Committee) with the decision dated 12/04/2023. It numbered 2023/139, and the study was conducted according to the ethical standards of the Declaration of Helsinki. Sociodemographic and clinical characteristics of all participants, depressive symptoms, and OCD severity level of the patients in the OCD group were evaluated with clinical scales. Venous blood samples were then collected from the participants.

## Measures

### Demographic and Clinical Data Form

This form was prepared by the clinicians involved in the study to assess the demographic and clinical characteristics of the adolescents. It was completed by the child psychiatrists involved in the study by interviewing the participants.

### Children’s Yale-Brown Obsessive–Compulsive Scale (CY-BOCS)

In children and adolescents with OCD, Scahill et al. have demonstrated the reliability and validity of the CY-BOCS subscales and total score (Scahill et al. 1997). The CY-BOCS is a 10-item, clinician-rated, semi-structured instrument designed to assess an individual’s OCD symptom severity in the previous week. This scale is completed and scored by the clinician in an interview with the patient. Each item related to the severity of obsessions and compulsions is rated from 0 (none) to 4 (severe), and the total score ranges from 0 to 40. It was found to be a valid and reliable instrument for OCD severity in the Turkish population (adolescents) (Yucelen et al. 2006).

### DSM-5 Level 2 Depression Scale-Turkish Version (Child Form for 11–17 Years (DDS-CF)

The DDS-CF is a 14-item self-report scale that assesses depressive symptoms in children and adolescents and is completed by the adolescent (American Psychiatric Association 2013). The minimum score obtained from the scale is 14, and the maximum score is 70. In the reliability analysis of the DDS-CF in the Turkish population, Cronbach’s alpha internal consistency coefficient was found to be 0.96 for the child form, and it was stated that the items in the scale were consistent with each other, and the reliability of the measurement tool was high (SAPMAZ et al. 2017).

### Assessment of Biochemical Parameters

Venous blood samples were collected from the participants between 08.00 and 09.30 in the morning after 12 h of fasting. Blood samples from each patient were placed in a biochemistry tube and centrifuged at 4000 rpm for 10 min after a half-hour incubation period. After centrifugation, the participants’ sera were separated, and the serum samples were stored at − 80 °C until the study. On the study day, serum samples were thawed, and EPO and IMA were analyzed. EPO levels in serum samples were studied using the automated solid phase chemiluminescence immunometric method (Immulite 2000, Siemens, USA). In the data provided by the manufacturer, the within-study coefficients of variability ranged from 5.2 to 5.6% for the concentration range of 4.1–184 mlU/ml and from 5.8 to 5.9% overall. The analytical sensitivity was reported as 1.0 mlU/ml, and the upper limit of calibration was 200 mlU/ml. IMA levels were determined by colorimetric method using ready-made commercial kits in an auto-analyzer device (Beckman AU-5600, USA). In this method, normal albumin is present in serum or plasma as the active form. When adding cobalt chloride solution, the N-terminus of albumin combines with Co + , and the concentration of free Co + in the solution decreases. At higher levels of IMA, the ability of albumin to bind Co + is reduced when adding cobalt chloride solution, so the free Co + concentration is higher. The higher concentration of free Co + produces more colored products when dithiothreitol (DTT) is added. In a colorimetric test at a given wavelength, the absorbance is proportional to the free Co + concentration. The IMA concentration in the sample is calculated by comparing the same process with the calibrator result. The sample’s absorbance change is directly proportional to the IMA level. The absorbance of the samples was measured at 470 nm using a spectrophotometer, and IMA levels were expressed in absorbance units (ABSU). Albumin levels were determined by an autoanalyzer (Olympus AU-5600) at the center where the study was performed and expressed as g/L.

### Statistical Analysis

SPSS 23.0 program was used for statistical analysis, and *p* < 0.05 was considered statistically significant. The normal distribution of the data was analyzed based on skewness and kurtosis values. Skewness and kurtosis values between + 1.5 and − 1.5 were considered normal distributions. IMA values were normally distributed in both OCD and control groups. EPO values were normally distributed in the control group but not in the OCD group. Clinical scale scores (CY-BOCS, DDS-CF) were found to be normally distributed. Mann–Whitney U test was used for the differences between EPO levels, and the independent sample *t*-test was used for the differences between IMA levels between the two groups. Associations between categorical variables were evaluated using the chi-square test and Fisher’s exact test. The relationship between EPO and OCD severity and disease duration was analyzed by Spearman’s correlation, and the relationship between IMA and OCD severity and disease duration was analyzed by Pearson’s correlation. In the study, the researchers utilized receiver operating characteristic (ROC) Analysis to identify the optimal cut-off points for distinguishing between groups based on EPO and IMA parameters and calculated the area under the curve (AUC) to assess the model’s accuracy. The Youden Index was employed to determine the ROC cut-off values, providing a comprehensive approach for determining the discriminatory ability of the EPO and IMA parameters.

## Results

Sixty-eight adolescents, 35 (51.5%) patients in the OCD group, and 33 (48.5%) participants in the healthy control group participated in the study. The mean age of the OCD group was 14.94 ± 1.73 years, and the mean age of the healthy control group was 15.00 ± 1.58 years (*p* = 0.888, *t* =  − 0.142). In the OCD group, 18 (51.4%) were male and 17 (48.6%) were female. In the healthy control group, 14 (42.4%) were male and 19 (57.6%) were female (*p* = 0.457). Out of the participants in the OCD group, 15 had at least one parent with a psychiatric disorder. In comparison, only 5 participants in the healthy control group had at least one parent with a psychiatric disorder (*p* = 0.012), as shown in Table 1. Twenty (57.1%) of the patients in the OCD group used psychotropic medication, while 15 (42.9%) did not use any medication. Among the patients who used psychotropic medication, 11 (55.0%) were on antidepressant medication only, while 9 (45.0%) were on a combination of antidepressants and antipsychotics. In addition, no significant statistical difference regarding BMI was found between the groups (*p* = 0.160, *t* = 1.422).

**Table 1:** Clinical and demographic features

Feature		Groups		*p*
		OCD group (*n* = 35)	HC group(*n* = 33)	
Age (year)	(Mean ± SD)	14.94 ± 1.73	15.00 ± 1.58	0.888
Gender	Male	18 (51.4%)	14 (42.4%)	0.457
	Female	17 (48.6%)	19 (57.6%)	
Psychiatric disorders in parents	Yes	15 (42.8%)	5 (15.2%)	0.012
	No	20 (57.2%)	28 (84.8%)	
Mothers’ occupational status	Yes	11 (31.4%)	10 (30.3%)	0.920
	No	24 (68.6%)	23 (69.7%)	
Father’s occupational status	Yes	29 (88.6%)	28 (68.1%)	0.812
	No	4 (11.4%)	3 (3.1%)	
BMI (kg/m^2^)	(Mean ± SD)	20.38 ± 2.34	19.70 ± 1.58	0.160
Duration of OCD (month)	(Mean ± SD)	19.08 ± 7.62	-	
Use of psychotropic drugs (yes/no)	Yes	20 (57.1%)	-	
	No	15 (42.9%)		
Duration of medication treatment (month)	Median [min–max]	6 (1–24)	-	
CY-BOCS total score	(Mean ± SD)	25.11 ± 6.46	-	
CY-BOCS-obsession	(Mean ± SD)	12.40 ± 3.22	-	
CY-BOCS-compulsion	(Mean ± SD)	12.71 ± 3.35	-	
DDS-CF	(Mean ± SD)	24.31 ± 3.84	17.57 ± 1.65	< 0.001

Abbreviations: *OCD* obsessive-compulsive disorder, *HC* healthy control, *CY-BOCS* Children’s Yale-Brown Obsessive-Compulsive Scale, *DDS-CF* DSM-5 Level 2 Depression Scale-Child Form

The most common obsessions in the OCD group were contamination obsessions in 19 (54.3%) patients, magical thoughts/superstitious belief obsessions in 12 (34.3%) patients, somatic obsessions in 10 (28.6%) patients, aggression obsessions in 8 (22.9%) patients, and sexual obsessions in 7 (20.0%) patients. The least common obsessions were religious obsessions in 6 (17.1%) patients, other obsessions (need to know and remember, fear of not saying exactly the right thing) in 4 (11.4%) patients, and hoarding/storing obsessions in 3 (8.6%) patients. The most common compulsions were washing/cleaning compulsions in 19 patients (54.3%), controlling compulsions in 17 patients (48.6%), other compulsions in 9 patients (25.7%), excessive magical thoughts/superstitious behaviors in 7 patients (20.0%), repetitive ritual behaviors in 7 patients (20.0%), and ritual behaviors involving other people in 6 patients (17.1%). The least common compulsions were counting in 5 (14.3%) patients, sorting/organizing in 4 (11.4%) patients, and accumulation/collection in 3 (8.6%) patients, respectively.

The CY-BOCS-total score was 25.11 ± 6.46, the CY-BOCS-obsession score was 12.40 ± 3.22, and the CY-BOCS-compulsion score was 12.71 ± 3.35 in the OCD group. The DDS-CF scores of the OCD group were significantly higher than the healthy control group (*p* < 0.001). The CY-BOCS total scores of the patients in the OCD group were between 15 and 38 points. Two (5.7%) patients had a CY-BOCS total score of 15 (mild). Thirteen (37.2%) patients had a CY-BOCS-total score between 16 and 23 (moderate), 14 (40.0%) patients had a score between 24 and 31 (severe), and 6 (17.1%) patients had a score of 32 points or more (extreme).

**Fig. 1:**
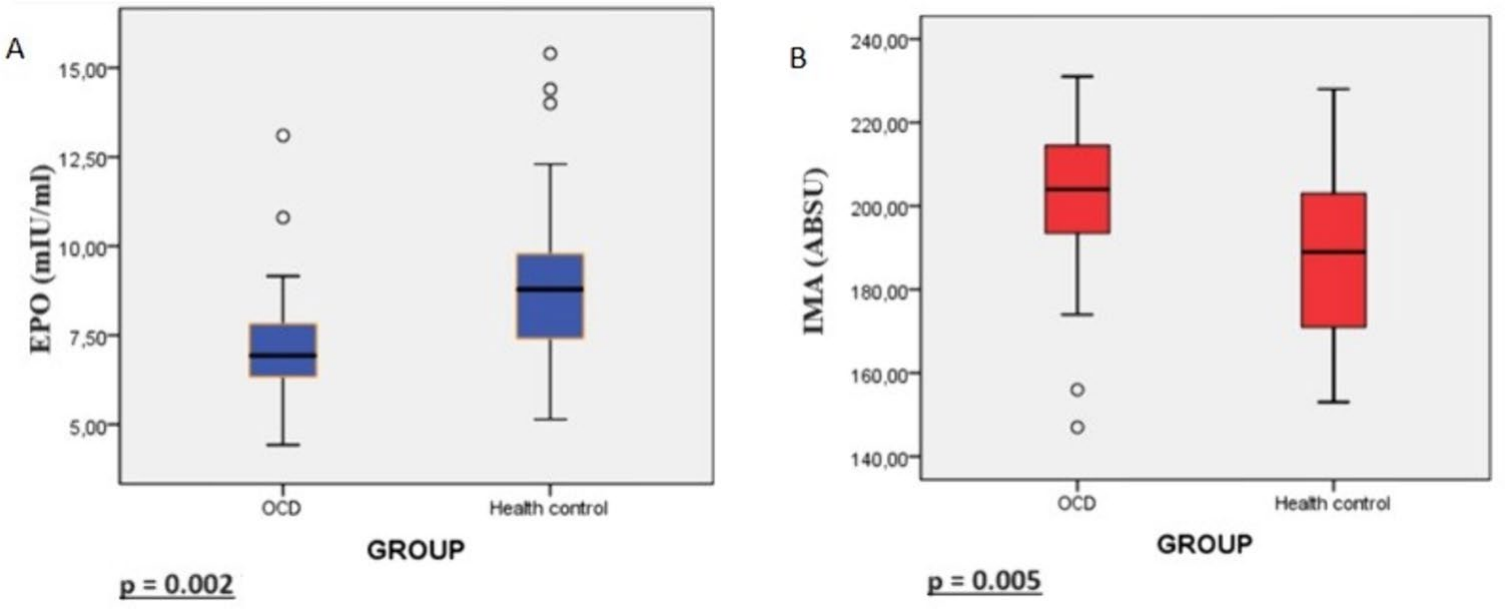
**A** Comparison of serum EPO levels between groups. **B** Comparison of serum IMA levels between groups

The median values of EPO levels were 6.93 mIU/ml and 8.79 mIU/ml in OCD and healthy control groups, respectively. EPO levels were lower in OCD patients compared to healthy controls (*p* = 0.002; *Z* =  − 3.123) (Fig. 1A). In the OCD group, the lowest EPO value was 4.43 mIU/ml, and the highest EPO value was 13.10 mIU/ml. The lowest EPO value in healthy controls was 5.14 mIU/ml, and the highest was 15.40 mIU/ml. Serum IMA levels (ABSU) were 202.14 ± 19.04 in the OCD group and 187.78 ± 21.64 in the healthy control group, and IMA levels were significantly higher in the OCD group (*p* = 0.005, *t* = 2.908) (Fig. 1B). Albumin levels were 44.91 ± 2.59 in the OCD group and 44.78 ± 2.28 in the control group (*p* = 0.828, *t* = 0.219).

There was no significant difference in serum EPO and IMA levels between OCD patients on psychotropic medication and OCD patients without psychotropic medication (*p* = 0.726, *Z* =  − 0.350; *p* = 0.463, *Z* =  − 0.734, respectively).

**Fig. 2:**
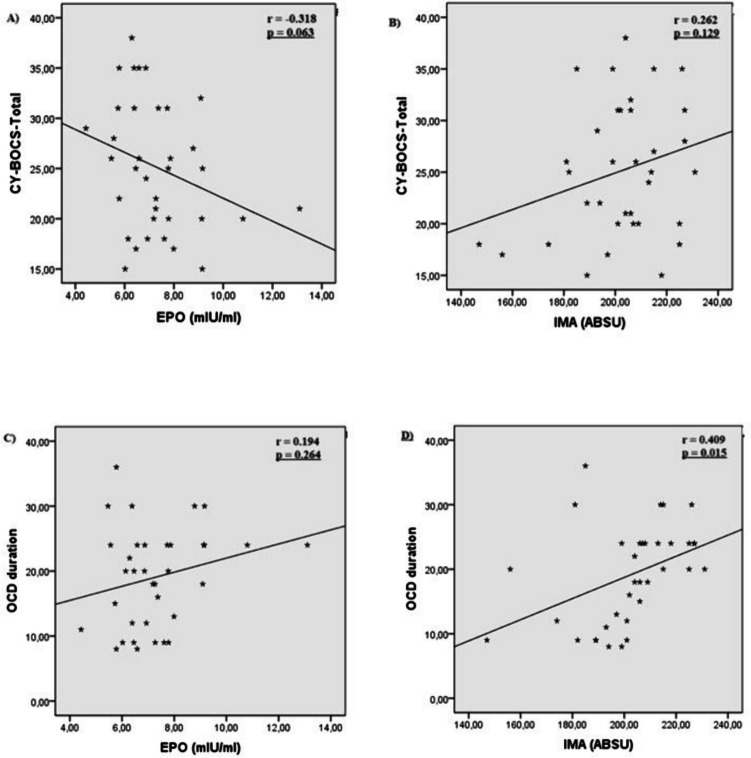
**A** Correlation between EPO levels and CY-BOCS-total scores in OCD group. **B** Correlation between IMA levels and CY-BOCS-total scores in OCD group. **C** Correlation between EPO levels and duration of disease in OCD group. **D** Correlation between IMA levels and duration of disease in OCD group

There was no significant correlation between serum EPO and IMA levels and OCD severity (*r* =  − 0.318, *p* = 0.063 and *r* = 0.262, *p* = 0.111, respectively) (Fig. 2A, B). No significant correlation was found between EPO levels and the duration of OCD (*r* = 0.194, *p* = 0.264) (Fig. 2C). A significant positive correlation was found between IMA levels and the duration of OCD symptoms (*p* = 0.015, *r* = 0.409) (Fig. 2D) (Table 2).

**Table 2:** Association of biochemical markers with disease severity and duration

		CY-BOCS-obsession severity	CY-BOCS-compulsion severity	CY-BOCS-total score	OCD duration (month)
EPO	r*	− 0.299	− 0.301	− 0.318	0.194
	*p*	0.081	0.079	0.063	0.264
IMA	r**	0.239	0.274	0.262	0.409
	*p*	0.167	0.111	0.129	0.015

Abbreviations: *EPO* erythropoietin, *IMA* ischemic-modified albümin, *OCD* obsessive-compulsive disorder, *CY-BOCS* Children’s Yale-Brown Obsessive-Compulsive Scale

^*^Spearman correlation coefficient

^**^Pearson correlation coefficient

**Fig. 3:**
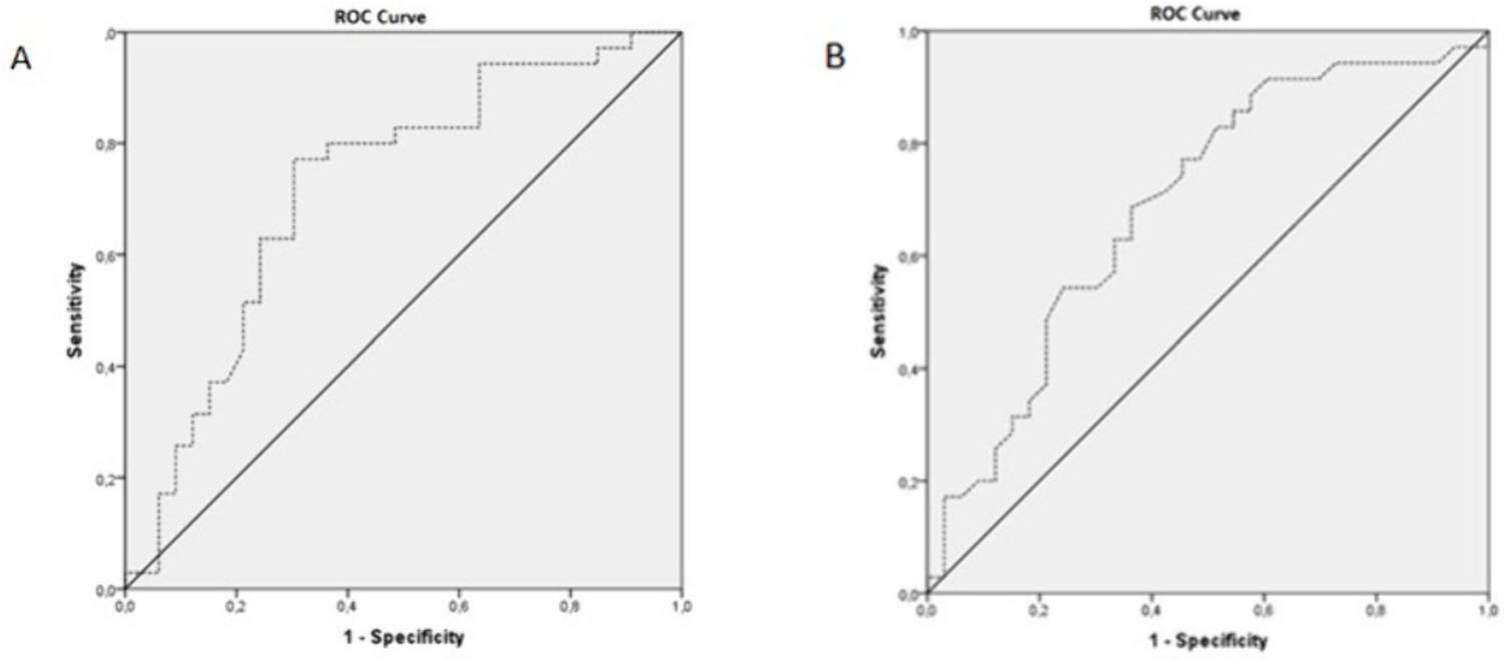
**A** Discriminative capacity of EPO levels to distinguish between OCD group and healthy controls. **B** The discriminative capacity of IMA levels between OCD group and healthy controls

ROC curve was drawn for EPO (Fig. 3A) and IMA (Fig. 3B) levels, and the AUC was 0.692 (*p* = 0.007) for IMA and 0.720 (*p* = 0.002) for EPO. The cut-off point for IMA levels was 198.0 (sensitivity: 68.6%, specificity: 63.6%), and the cut-off point for EPO levels was 7.39 (sensitivity: 62.9%, specificity: 75.8%) (Table 3).

**Table 3:** Cut-off values for EPO and IMA parameters in adolescents with obsessive–compulsive disorder

**EPO**	0.720	7.39	62.9%	75.8%	**0.002**	0.596	0.845
IMA	0.692	198.0	68.6%	63.6%	**0.007**	0.565	0.819

Abbreviations: *AUC* area under the curve, *EPO* erythropoietin, *IMA* ischemia‑modified albumin

## Discussion

The relationship between oxidative systems and obsessive–compulsive disorder (OCD) has been a topic of interest in the field of psychiatry and neuroscience. In order to better understand the connection between OS and OCD, it is important to examine the molecular and cellular mechanisms that contribute to oxidative stress and its potential role in the neurobiology of OCD. The findings of this study show that EPO levels, which have an antioxidant effect, are significantly lower, and IMA levels, one of the indicators of OS, are significantly higher in OCD patients compared to the control group. This suggests that inflammatory and oxidative processes may play a role in the pathogenesis of OCD. Furthermore, the study revealed a positive correlation between OCD duration and IMA levels. Additionally, the AUCs obtained from the ROC analysis suggest that EPO and IMA levels may serve as potential biomarkers for distinguishing adolescents with OCD from healthy controls.

OS can lead to the accumulation of ROS in the central nervous system, which can cause damage to neurons and impair their function. This can result in neuroinflammation, synaptic dysfunction, and alterations in neurotransmitter levels, all of which are implicated in the development and progression of psychiatric conditions (Smaga et al. 2021). The brain’s high content of unsaturated fatty acids, high oxygen consumption, and low levels of antioxidant defense systems make it highly susceptible to oxidative damage. Additionally, the brain’s vulnerability to OS is further exacerbated by the high oxygen consumption and the susceptibility of pericytes in the brain microvessels to OS, which is crucial for maintaining the integrity of the blood–brain barrier (Hurtado-Alvarado et al. 2014; Li et al. 2022).

In this study, EPO levels were found to be lower in OCD patients compared to the control group, and EPO levels may be an indicator of OS in OCD. While various inflammatory and oxidative stress markers have been extensively examined in OCD through clinical studies (Danışman Sonkurt et al. 2022) and meta-analyses (Maia et al. 2019), to our knowledge, there has been no research conducted on the levels of erythropoietin (EPO) in OCD patients. EPO’s role within the brain is to inhibit nitric oxide, activate antioxidant enzymes, prevent the apoptotic process, reduce excitotoxicity, and inhibit inflammation (Oorschot et al. 2020). When EPO is routinely administered at appropriate doses, it has been shown to cross the blood–brain barrier and increase its neuroprotective effects (Fond et al. 2012). Due to EPO’s activity on the CNS, EPO levels or the efficacy of EPO treatment has been the subject of research, especially in psychiatric disorders. EPO treatment has been investigated in human studies in psychiatric disorders such as treatment-resistant depression, bipolar disorder, and schizophrenia and in an animal model of autism spectrum disorder (Miskowiak et al. 2014; Ott et al. 2016; Petersen et al. 2018; Solmaz et al. 2020). The findings obtained in these studies show that EPO has positive effects on cognitive functions, executive functions, quality of life, and memory. However, in studies conducted with peripheral EPO levels, it was found that plasma EPO levels were low in ADHD patients, and plasma EPO levels in MDD patients were significantly associated with suicidality (Gungor et al. 2021; Lee et al. 2021). In another study conducted in children with ADHD, plasma EPO levels did not differ from healthy controls, but there was a significant relationship between EPO levels and ADHD severity. The authors stated that this may be a compensatory mechanism against oxidative damage (Shim et al. 2021). The low serum EPO levels observed in OCD patients are consistent with previous studies in psychiatric and neurodegenerative disorders. These findings suggest that EPO may contribute to the development of oxidative and inflammatory processes occurring in OCD, but this requires further investigation.

The findings of the present study support that EPO may be a diagnostic biomarker in OCD based on a cut-off point of 7.39 mlU/mol (AUC: 0.720; sensitivity: 62.9%; specificity: 75.8%). The diagnostic usefulness of EPO levels has been investigated in children with ADHD and adults with generalized anxiety disorder (GAD) (Gungor et al. 2021; Kurutas 2023). EPO levels were found to be lower in patients with GAD, and the sensitivity and specificity of serum EPO levels were found to be 100% and 85.9%, respectively, based on a cut-off point of 6.11 mlU/mol (AUC: 0.901) (Kurutas 2023). Similarly, it was reported that serum EPO levels were lower in children with ADHD, the sensitivity was 100%, and specificity was 97.14% based on a cut-off point of 6.27 mlU/mol (AUC = 0.980) (Gungor et al. 2021). Consistent with studies in other psychiatric disorders, our study provides evidence supporting the potential use of EPO levels as a diagnostic biomarker in OCD. However, it is essential to consider the broader clinical context when interpreting these findings.

Another finding of this study revealed that patients with OCD had higher levels of IMA, which is an indicator of OS, compared to the control group. Furthermore, the duration of OCD was positively associated with IMA levels. IMA is a product of protein oxidation, and recent evidence suggests that IMA can be used as an indicator of oxidative damage (Tampa et al. 2022). There is evidence that serum IMA levels are a more reliable marker than total antioxidant capacity and oxidative stress index in determining the level of OS (KESKİN et al. 2019). IMA levels have been investigated in the field of psychiatric disorders in recent years, especially in major psychiatric disorders, and there are limited studies in the pediatric age group. In a study involving adults diagnosed with major depressive disorder, it was observed that serum IMA levels were elevated. Additionally, there was a significant positive correlation between the severity of depression and IMA levels (Karaaslan et al. 2019). Although IMA levels in patients with schizophrenia did not differ significantly from healthy controls, the authors interpreted that IMA levels tended to increase (Tunç et al. 2019). However, it was reported that IMA levels in patients with bipolar disorder did not differ from healthy controls during the remission period, and IMA levels measured during acute manic episodes were higher than controls (Korkmaz et al 2023). To our knowledge, IMA levels have only been studied in autism spectrum disorder in children with psychiatric disorders. It was reported that serum IMA levels were higher in patients with autism compared to controls, and there was a significant positive correlation between IMA levels and myeloperoxidase (Ceylan et al. 2021). No significant correlation was found between IMA levels and disease severity in studies conducted with patients with both manic episodes and autism (Ceylan et al. 2021; Korkmaz et al 2023). In summary, elevated serum IMA levels in OCD patients are consistent with previous studies in psychiatric disorders. These results support that IMA, an indicator of OS, may play a role in the etiopathogenesis of OCD and that IMA levels can be used as an indicator of previously documented OS in OCD.

The results of the ROC analysis indicate that IMA levels can discriminate adolescents with OCD from controls with 68.6% sensitivity and 63.6% specificity and may have diagnostic significance (AUC: 0.692; cut-off value: 198.0; 95% CI: 0.565–0.0.819). To the authors’ knowledge, no study has investigated the diagnostic validity of IMA levels in OCD and other psychiatric disorders. The diagnostic validity of IMA levels has been investigated in brucellosis, ischemic stroke, and chronic liver diseases (Ahn et al. 2011; Dündar 2023; Yavuz et al. 2017). In these disorders, the AUC was found in a wide range of 0.560 to 0.990. Although our results provide promising findings, we do not consider our results as a new biomarker since there is no similar study in psychiatric disorders. Further research on the diagnostic validity of IMA levels in OCD may clarify this issue.

Thiol/disulfide parameters, an indicator of OS in adolescents, were positively correlated with the duration of OCD (Ozkan et al. 2021). Another finding of the present study was that serum IMA levels showed a moderate positive correlation with the duration of OCD. There is substantial evidence suggesting that low-grade inflammation and oxidative processes are prevalent early in life in individuals with OCD (Gerentes et al. 2019). These findings underscore the significance of early detection and intervention for OCD, as delayed diagnosis may exacerbate the severity of the disorder and OS.

A small number of patients and a cross-sectional design were significant limitations of the present study; therefore, generalization of the results may not be possible. EPO and IMA levels were assessed using serum samples. The extent to which the levels of these markers in serum correlate with their levels in the brain is still unclear. Considering the prevalence of OCD in both pre-adolescence and adulthood, another limitation of our study is that the participants were in adolescence. In addition, EPO and IMA levels may be affected by the menstrual cycle in women. This has not been taken into account in female patients, and it is important to consider this in future studies. Although we controlled for some factors, we did not consider factors affecting OS, such as diet, lifestyle, exercise, or activity level. In addition, other environmental, psychosocial stress, and genetic factors that may increase OS were not considered.

## Conclusion

In conclusion, the study’s findings contribute to the growing body of evidence implicating inflammatory and oxidative processes in the pathogenesis of OCD. The potential of EPO and IMA levels as diagnostic biomarkers for OCD aligns with the ongoing efforts to identify reliable biological markers for the disorder. EPO therapy and antioxidant treatment options may be an effective treatment option for OCD patients. Further studies on larger samples will help determine whether EPO and IMA levels have a direct effect on the etiopathogenesis of OCD. Further research in this area is warranted to validate the utility of IMA and EPO levels as a biomarker for OCD.

## Data Availability

The datasets analyzed during the current study are available from the corresponding author upon reasonable request.

## References

[CR1] Ahn JH, Choi SC, Lee WG, Jung YS (2011) The usefulness of albumin-adjusted ischemia-modified albumin index as early detecting marker for ischemic stroke. Neurol Sci 32(1):133–138 21153598 10.1007/s10072-010-0457-4

[CR2] Alici D, Bulbul F, Virit O, Unal A, Altindag A, Alpak G, Alici H, Ermis B, Orkmez M, Taysi S, Savas H (2016) Evaluation of oxidative metabolism and oxidative DNA damage in patients with obsessive-compulsive disorder. Psychiatry Clin Neurosci 70(2):109–115 26388322 10.1111/pcn.12362

[CR3] Alsheikh AM, Alsheikh MM (2021) Obsessive-compulsive disorder with rheumatological and inflammatory diseases: a systematic review. Cureus 13(5):e14791 33954077 10.7759/cureus.14791 PMC8088283

[CR4] American Psychiatric Association (APA) (2013) DSM-5 level-2 depression scale-child form. American Psychiatric Association. http://www.psychiatry.org/practice/dsm/dsm5/online-assessment-measures. Accessed 13 Feb 2024

[CR5] Ayala A, Muñoz MF, Argüelles S (2014) Lipid peroxidation: production, metabolism, and signaling mechanisms of malondialdehyde and 4-hydroxy-2-nonenal. Oxid Med Cell Longev 2014:360438 24999379 10.1155/2014/360438 PMC4066722

[CR6] Ceylan MF, Tural Hesapcioglu S, Yavas CP, Senat A, Erel O (2021) Serum ischemia-modified albumin levels, myeloperoxidase activity and peripheral blood mononuclear cells in autism spectrum disorder (ASD). J Autism Dev Disord 51(7):2511–2517 33029667 10.1007/s10803-020-04740-9

[CR7] Cho SB, Eum WS, Shin MJ, Kwon HJ, Park JH, Choi YJ, Park J, Han KH, Kang JH, Kim DS, Cho SW, Kim DW, Choi SY (2019) Transduced Tat-aldose reductase protects hippocampal neuronal cells against oxidative stress-induced damage. Exp Neurobiol 28(5):612–627 31698553 10.5607/en.2019.28.5.612 PMC6844837

[CR8] Danışman Sonkurt M, Altınöz AE, Köşger F, Yiğitaslan S, Güleç G, Eşsizoğlu A (2022) Are there differences in oxidative stress and inflammatory processes between the autogenous and reactive subtypes of obsessive-compulsive disorder? A controlled cross-sectional study. Braz J Psychiatry 44(2):171–177 34190826 10.1590/1516-4446-2021-1740 PMC9041960

[CR9] Du L, Ma J, He D, Zhang X (2019) Serum ischaemia-modified albumin might be a potential biomarker for oxidative stress in amnestic mild cognitive impairment. Psychogeriatrics 19(2):150–156 30362220 10.1111/psyg.12377

[CR10] Dündar A (2023) Investigation of serum ischemic-modified albumin, galectin-3, paraoxonase-1, and myeloperoxidase activity levels in patients with acute brucellosis. Redox Rep 28(1):2289727 38054459 10.1080/13510002.2023.2289727 PMC11001275

[CR11] Fond G, Macgregor A, Attal J, Larue A, Brittner M, Ducasse D, Capdevielle D (2012) Treating patients with schizophrenia deficit with erythropoietin? Psychiatry Clin Neurosci 66(5):375–382 22725970 10.1111/j.1440-1819.2012.02359.x

[CR12] Gerentes M, Pelissolo A, Rajagopal K, Tamouza R, Hamdani N (2019) Obsessive-compulsive disorder: autoimmunity and neuroinflammation. Curr Psychiatry Rep 21(8):78 31367805 10.1007/s11920-019-1062-8

[CR13] Goodman WK, Storch EA, Sheth SA (2021) Harmonizing the neurobiology and treatment of obsessive-compulsive disorder. Am J Psychiatry 178(1):17–29 33384007 10.1176/appi.ajp.2020.20111601 PMC8091795

[CR14] Gungor M, Kurutas EB, Oner E, Unsal V, Altun H, Yalin AE, Yalin S, Bozkus O, Sahin N (2021) Diagnostic performance of erythropoietin and erythropoietin receptors levels in children with attention deficit hyperactivity disorder. Clin Psychopharmacol Neurosci 19(3):530–536 34294622 10.9758/cpn.2021.19.3.530 PMC8316662

[CR15] Hurtado-Alvarado G, Cabañas-Morales AM, Gómez-Gónzalez B (2014) Pericytes: brain-immune interface modulators. Front Integr Neurosci 7:80 24454281 10.3389/fnint.2013.00080 PMC3887314

[CR16] Juul SE, Comstock BA, Heagerty PJ, Mayock DE, Goodman AM, Hauge S, Gonzalez F, Wu YW (2018) High-dose erythropoietin for asphyxia and encephalopathy (HEAL): a randomized controlled trial - background, aims, and study protocol. Neonatology 113(4):331–338 29514165 10.1159/000486820 PMC5980685

[CR17] Kandemir H, Abuhandan M, Aksoy N, Savik E, Kaya C (2013) Oxidative imbalance in child and adolescent patients with obsessive compulsive disorder. J Psychiatr Res 47(11):1831–1834 24011862 10.1016/j.jpsychires.2013.08.010

[CR18] Karaaslan Ö, Hacimusalar Y, Amuk ÖC, Ceylan B (2019) Evaluation of ischemia modified albumin levels in major depression patients. J Surg Med 3(8):557–560

[CR19] Keskin S, Arica DA, Orem A, Akcan B, Mentese A, Bahadir S (2019) Ischemia modified albumin: a useful marker for increased oxidative stress in Behçet’s disease. Mucosa 2(1):19–27

[CR20] Korkmaz ŞA, Kızgın S, Oğuz EF, Neşelioğlu S, Erel Ö (2023) Thiol-disulphide homeostasis, ischemia-modified albumin, complete blood count-derived inflammatory markers and C-reactive protein from acute mania to early remission in bipolar disorder. J Affect Disord 339:426–434 37459969 10.1016/j.jad.2023.07.079

[CR21] Kurutas EB (2023) Erythropoietin and erythropoietin receptor levels and their diagnostic values in drug-naïve patients with generalized anxiety disorder. Clin Psychopharmacol Neurosci 21(2):288–295 37119221 10.9758/cpn.2023.21.2.288 PMC10157015

[CR22] Lai YF, Lin TY, Ho PK, Chen YH, Huang YC, Lu DW (2022) Erythropoietin in optic neuropathies: current future strategies for optic nerve protection and repair. Int J Mol Sci 23(13):7143. 10.3390/ijms23137143 35806148 10.3390/ijms23137143 PMC9267007

[CR23] Larpthaveesarp A, Pathipati P, Ostrin S, Rajah A, Ferriero D, Gonzalez FF (2021) Enhanced mesenchymal stromal cells or erythropoietin provide long-term functional benefit after neonatal stroke. Stroke 52(1):284–293 33349013 10.1161/STROKEAHA.120.031191 PMC7770074

[CR24] Lee BH, Park YM, Hwang JA, Kim YK (2021) Variable alterations in plasma erythropoietin and brain-derived neurotrophic factor levels in patients with major depressive disorder with and without a history of suicide attempt. Prog Neuropsychopharmacol Biol Psychiatry 110:110324 33857523 10.1016/j.pnpbp.2021.110324

[CR25] Li J, Jia B, Cheng Y, Song Y, Li Q, Luo C (2022) Targeting molecular mediators of ferroptosis and oxidative stress for neurological disorders. Oxid Med Cell Longev 2022:3999083 35910843 10.1155/2022/3999083 PMC9337979

[CR26] Maia A, Oliveira J, Lajnef M, Mallet L, Tamouza R, Leboyer M, Oliveira-Maia AJ (2019) Oxidative and nitrosative stress markers in obsessive-compulsive disorder: a systematic review and meta-analysis. Acta Psychiatr Scand 139(5):420–433 30873609 10.1111/acps.13026

[CR27] Miskowiak KW, Vinberg M, Christensen EM, Bukh JD, Harmer CJ, Ehrenreich H, Kessing LV (2014) Recombinant human erythropoietin for treating treatment-resistant depression: a double-blind, randomized, placebo-controlled phase 2 trial. Neuropsychopharmacology 39(6):1399–1408 24322509 10.1038/npp.2013.335 PMC3988543

[CR28] Oorschot DE, Sizemore RJ, Amer AR (2020) Treatment of neonatal hypoxic-ischemic encephalopathy with erythropoietin alone, and erythropoietin combined with hypothermia: history, current status, and future research. Int J Mol Sci 21(4):1487. 10.3390/ijms21041487 32098276 10.3390/ijms21041487 PMC7073127

[CR29] Ott CV, Vinberg M, Kessing LV, Miskowiak KW (2016) The effect of erythropoietin on cognition in affective disorders - associations with baseline deficits and change in subjective cognitive complaints. Eur Neuropsychopharmacol 26(8):1264–1273 27349944 10.1016/j.euroneuro.2016.05.009

[CR30] Ottolenghi S, Milano G, Cas MD, Findley TO, Paroni R, Corno AF (2021) Can erythropoietin reduce hypoxemic neurological damages in neonates with congenital heart defects? Front Pharmacol 12:770590 34912224 10.3389/fphar.2021.770590 PMC8666450

[CR31] Ozkan Y, Kandemir H, Yalın Sapmaz S, Taneli F, Ozdemir H, Gozaçanlar Ozkan O (2021) Thiol/disulfide homeostasis in medication-naive children and adolescents with obsessive-compulsive disorder. J Psychiatr Res 140:159–164 34116441 10.1016/j.jpsychires.2021.05.084

[CR32] Pauls DL, Abramovitch A, Rauch SL, Geller DA (2014) Obsessive-compulsive disorder: an integrative genetic and neurobiological perspective. Nat Rev Neurosci 15(6):410–424 24840803 10.1038/nrn3746

[CR33] Petersen JZ, Schmidt LS, Vinberg M, Jørgensen MB, Hageman I, Ehrenreich H, Knudsen GM, Kessing LV, Miskowiak KW (2018) Effects of recombinant human erythropoietin on cognition and neural activity in remitted patients with mood disorders and first-degree relatives of patients with psychiatric disorders: a study protocol for a randomized controlled trial. Trials 19(1):611 30400939 10.1186/s13063-018-2995-7 PMC6220567

[CR34] Sapmaz ŞY, Yalin N, Kavurma C, Tanriverdi BU, Öztekşn S, Köroğlu E, Aydemir Ö (2017) Reliability and validity of the DSM-5 level 2 Depression Scale-Turkish version (child form for 11–17 years and parent form for 6–17 years). J Cogn-Behav Psychother Res 6(1):15–15

[CR35] Scahill L, Riddle MA, McSwiggin-Hardin M, Ort SI, King RA, Goodman WK, Cicchetti D, Leckman JF (1997) Children’s Yale-brown obsessive compulsive scale: reliability and validity. J Am Acad Child Adolesc Psychiatry 36(6):844–852 9183141 10.1097/00004583-199706000-00023

[CR36] Seshadri Reddy V, Duggina P, Vedhantam M, Manne M, Varma N, Nagaram S (2018) Maternal serum and fetal cord-blood ischemia-modified albumin concentrations in normal pregnancy and preeclampsia: a systematic review and meta-analysis. J Matern Fetal Neonatal Med 31(24):3255–3266 28817994 10.1080/14767058.2017.1368480

[CR37] Shevtsova A, Gordiienko I, Tkachenko V, Ushakova G (2021) Ischemia-modified albumin: origins and clinical implications. Dis Markers 2021:9945424 34336009 10.1155/2021/9945424 PMC8315882

[CR38] Shim SH, Kim YK, Hwangbo Y, Yoon HJ, Kim JS, Lee YJ, Woo YS, Bahk WM (2021) The relationship between plasma erythropoietin levels and symptoms of attention deficit hyperactivity disorder. Clin Psychopharmacol Neurosci 19(2):334–340 33888662 10.9758/cpn.2021.19.2.334 PMC8077052

[CR39] Smaga I, Frankowska M, Filip M (2021) N-acetylcysteine as a new prominent approach for treating psychiatric disorders. Br J Pharmacol 178(13):2569–2594 33760228 10.1111/bph.15456

[CR40] Solmaz V, Erdoğan MA, Alnak A, Meral A, Erbaş O (2020) Erythropoietin shows gender dependent positive effects on social deficits, learning/memory impairments, neuronal loss and neuroinflammation in the lipopolysaccharide induced rat model of autism. Neuropeptides 83:102073 32736811 10.1016/j.npep.2020.102073

[CR41] Stein DJ, Costa DL, Lochner C, Miguel EC, Reddy YJ, Shavitt RG, van den Heuvel OA, Simpson HB (2019) Obsessive–compulsive disorder. Nat Rev Dis Primers 5(1):52 31371720 10.1038/s41572-019-0102-3 PMC7370844

[CR42] Tampa M, Mitran CI, Mitran MI, Amuzescu A, Matei C, Georgescu SR (2022) Ischemia-modified albumin-a potential new marker of oxidative stress in dermatological diseases. Medicina (Kaunas) 58(5):669. 10.3390/medicina58050669 35630086 10.3390/medicina58050669 PMC9147831

[CR43] Tunç S, Atagün Mİ, Neşelioğlu S, Bilgin YY, Başbuğ HS, Erel Ö (2019) Ischemia-modified albumin: a unique marker of global metabolic risk in schizophrenia and mood disorders. Psychiatry and Clinical Psychopharmacology 29(2):123–129

[CR44] Ünal F, Öktem F, Çetin Çuhadaroğlu F, Çengel Kültür SE, Akdemir D, Foto Özdemir D, Çak HT, Ünal D, Tıraş K, Aslan C, Kalaycı BM, Aydos BS, Kütük F, Taşyürek E, Karaokur R, Karabucak B, Karakök B, Karaer Y, Artık A (2019) Reliability and validity of the schedule for affective disorders and schizophrenia for school-age children-present and lifetime version, DSM-5 November 2016-Turkish Adaptation (K-SADS-PL-DSM-5-T). Turk Psikiyatri Derg 30(1):42–50 31170306

[CR45] Vittori DC, Chamorro ME, Hernández YV, Maltaneri RE, Nesse AB (2021) Erythropoietin and derivatives: potential beneficial effects on the brain. J Neurochem 158(5):1032–1057 34278579 10.1111/jnc.15475

[CR46] Yavuz F, Biyik M, Asil M, Dertli R, Demir A, Polat H, Uysal S, Ataseven H (2017) Serum ischemic modified albumin (IMA) concentration and IMA/albumin ratio in patients with hepatitis B-related chronic liver diseases. Turk J Med Sci 47(3):947–953 28618749 10.3906/sag-1611-66

[CR47] Yucelen AG, Rodopman-Arman A, Topcuoglu V, Yazgan MY, Fisek G (2006) Interrater reliability and clinical efficacy of Children’s Yale-brown obsessive-compulsive scale in an outpatient setting. Compr Psychiatry 47(1):48–53 16324902 10.1016/j.comppsych.2005.04.005

